# Knowledge and Attitude of Lebanese Women Regarding Induced Abortion: A Cross‐Sectional Study

**DOI:** 10.1002/hsr2.73027

**Published:** 2026-08-11

**Authors:** Youssef Abou Dib, Rayyan Hammoud, Sara Al Smaily, Tarek Abdul Samad, Nour Mina, Aline Bou Dargham, Alaa Abou Saleh, Bilal Azakir

**Affiliations:** ^1^ Faculty of Medicine Beirut Arab University Beirut Lebanon

**Keywords:** abortion, knowledge, Lebanon, perception, women's health

## Abstract

**Background and Aims:**

Induced abortion is a sensitive public health concern in Lebanon, particularly with the existence of Legal restriction and informal access to abortion services. Data regarding knowledge, attitudes, and practices of induced abortion among Lebanese women is limited, thus, this study aimed to assess knowledge, attitudes and practices and to identify the most common associated factors.

**Methods:**

This is a cross‐sectional study conducted among 502 Lebanese women aged over 18 years. An online self‐administered survey was distributed among the different governorates in Lebanon to assess sociodemographic characteristics, knowledge, attitudes, and practices of induced abortion. Bivariate and multivariate logistic regression models were used to determine factors associated with the outcomes.

**Results:**

The mean knowledge score was 9.76 ± 1.71 out of 14. Most of participants were unaware of the Lebanese abortion law, 61.8% believed abortion should not be socially acceptable, and 9% of participants reported having undergone an induced abortion and the majority used safe methods.

**Conclusion:**

Lebanese women demonstrated moderate knowledge about abortion but substantial gaps in legal awareness and knowledge of medication abortion were identified. Findings highlight the importance of a culturally appropriate reproductive health awareness and educational programs to promote abortion safety within the context of the Lebanese law restrictions.

## 1 Introduction

Globally, 73 million induced abortions occur annually according to the World Health Organization (WHO) [1]. Despite these high numbers, abortion remains an issue of political discourse. As of June 2022, access to abortion is no longer protected in the United States. The decision left it to each state to either outlaw abortion or make it more accessible [2]. This change sparked renewed global debates on abortion, with various public figures voicing their opposition or support for abortion bans. Nonetheless, the International Federation of Gynecology and Obstetrics (FIGO) is very clear on this topic, and it advocates for global access to safe abortion as a basic human right [3].

Abortion refers to the termination of a pregnancy before 20 weeks of gestation, and it may either be spontaneous or induced [4, 5, 6]. If induced abortion is performed according to medical standards, it is considered a safe abortion, and unsafe if otherwise [7].

In developing countries with restrictive laws, unsafe abortions remain highly prevalent (45%) [1, 8]. Unwanted pregnancies, improper use of contraceptive methods, and their limited accessibility are reported as the most common reasons for induced abortion [7, 9]. In fact, the practice of unsafe abortion is related to inadequate resources, cultural norms and stigma, and a low socioeconomic status [10]. According to the Guttmacher Institute, an estimated 22,000 women die annually from the complications of unsafe abortion [11] which include hemorrhage and infections [10, 12]. Therefore, post‐abortion care is necessary for the treatment of women who undergo unsafe or incomplete abortion [12].

Several studies on the knowledge, attitude, and practice of abortion have been conducted worldwide. Abortion misinformation, unfamiliarity with national laws, and religious and cultural restrictions were reported in Turkey [13]. In Ethiopia, two‐thirds of women of reproductive age had poor knowledge and negative attitude toward induced abortion [14]. A systemic review concluded that women have limited knowledge on the legality of abortion in their countries [15]. These studies reflected the lack of knowledge, the negative attitude, and the limited access to safe abortion.

In Lebanon, abortion remains legally restricted under articles 539–546 of the Penal Code, dating back to 1943. These laws were revised and amended in 1969 to allow abortion only as a last resort to save a woman's life following the approval of two physicians other than the attending one. Any termination outside this narrow legal scope subjects both the woman and her physician to imprisonment [16]. Despite legal restrictions, women in Lebanon can still informally access abortion services. Previous research described physicians as “moral gatekeepers” because eligibility for abortion remains related to their individual judgements [17]. Yet, accurate national reports on the number of induced abortions in Lebanon remain unavailable [16].

Given the limited data on Lebanese women's knowledge on abortion, attitudes toward abortion, and abortion‐related practices, this study aimed to explore these dimensions and their associated sociodemographic factors. Findings may contribute to the development of more appropriate, tailored, and targeted reproductive health educational programs and public health plans within the context of Lebanon's cultural variety and restrictive legal context.

## 2 Methodology

### 2.1 Study Design and Settings

This is a cross‐sectional study conducted across all Lebanese governorates. The data were collected using an online self‐administered survey by convenience sampling across all Lebanese governorates between June 2024 and January 2025. Participants were Lebanese women aged 18 and above who could read and provide informed consent. No additional exclusion criteria were applied.

### 2.2 Sample Size and Sampling Procedure

The Lebanese population is estimated around 5 million. With a confidence level of 95%, and a 5% margin of error, the minimal sample size needed was estimated to be 385 participants. Five hundred two women were collected by convenience covering the Lebanese population between the five governorates. Although the sample was non‐probabilistic, the geographic distribution of the participants was monitored to guarantee all governorates coverage.

### 2.3 Survey

Data was collected using an online self‐administered survey that was adopted from a previous study [18]. Questions and variables were reviewed by experts in the field and adapted to the Lebanese sociocultural context and legal regulations. The survey was originally developed in English and then translated into Arabic using a forward–backward translation approach to ensure consistency and comprehensibility.

The survey was designed on Google Forms and distributed using social media platforms. A pilot study was conducted among 25 participants to assess the comprehension and the understanding of the study and the reliability of the knowledge score. For the knowledge score, Each correct question was given one grade for a maximum of 14. The knowledge score demonstrated an acceptable internal consistency with a Cronbach's alpha of 0.74. Based on the pilot feedback, minor wording was adjusted before the final distribution.

The survey consisted of five sections: introduction and consent, sociodemographic data (age, place of residence, marital status, having children, occupation, educational level, being in the medical field, socioeconomic status, and the religious beliefs), knowledge assessment, attitude assessment, and practice of abortion.

### 2.4 Data Analysis

Data were analyzed using IBM SPSS Statistics for Windows, Version 25.0 (IBM Corp., Armonk, NY, USA). Categorical variables are presented as frequencies and percentages and the numerical variables as mean and standard deviation. The primary analyses focused on identifying factors independently associated with the main study outcomes using multivariate logistic regression models. Data were normally distributed, thus, parametric tests performed were Student's *t*‐test and analysis of variance (ANOVA) for continuous outcomes, and Pearson's chi‐square or Fisher's exact tests for categorical outcomes, as appropriate. The associations between continuous variables were evaluated using Pearson correlation. Variables with statistically significant association identified by the bivariate analysis were subsequently computed in multivariate logistic regression models to determine independent predictors for each outcome studied. For each outcome, adjusted odds ratios, 95% confidence intervals (CIs), and *p* values were reported. All statistical tests were two‐sided, and *p*‐value ≤ 0.05 was considered significant. Effect sizes are reported as adjusted odds ratios (ORs) with corresponding 95% confidence intervals to facilitate interpretation of the magnitude and direction of associations.

### 2.5 Ethical Consideration

This study was approved by the Institutional Review Board at Beirut Arab University under the number 2024‐H‐0144‐M‐R‐0586. All the data was collected anonymously; no names or identifiers were requested from the participants to be included in the survey. The objectives of this study were clear, and participation was completely voluntary. A consent form had to be agreed upon before participants started to fill the survey.

## 3 Results

The mean age of participants was 29.68 ± 10.63. The majority (83.5%) of them had university education, and almost 63.5% of them in non‐medical field. Most of the participants consider themselves as middle class and 66% of them considered themselves to be religious (Table 1).

**TABLE 1:** Basic demographic data of participants. SD represents the standard deviation.

Characteristics (502 participants)	Mean ± SD	
Age	29.68 ± 10.63	
	Number (*n*)	Percentage (%)
Marital status		
Single, never been in a relationship.	110	21.9
Single, no current relationship.	84	16.7
In a relationship	55	11.0
Married	232	46.2
Divorced	17	3.4
Widowed	4	0.8
Children (452)		
Yes	225	44.8
No	227	55.2
Residency		
North Lebanon	111	22.1
Mount Lebanon	173	34.5
Beqaa	73	14.5
South Lebanon	98	19.5
Beirut	47	9.4
Region		
Urban	207	41.2
Rural	295	58.8
Level of education Middle school		
High school	26	5.1
University	57	11.4
	419	83.5
Field of education (419)		
Medical	153	36.5
Non‐medical	266	63.5
Occupation		
Employed	191	38.0
Student	181	36.1
Non‐employed	130	25.9
Work field (181)		
Medical	40	22.1
Non‐medical	141	77.9
Mother's educational status		
Uneducated	35	7.0
Elementary school	318	63.3
Middle school	25	5.0
High school	44	8.8
University and HE	80	15.9
Father's educational status		
Uneducated	38	7.6
Elementary school	121	24.1
Middle school	116	23.1
High school	98	19.5
University and HE	129	25.7
Socioeconomic status		
Low class	37	7.4
Middle class	446	88.8
High class	19	3.8
Religious status		
Religious	332	66.1
Not religious	28	5.6
Religious to a certain extent	142	28.3

Among participants, 63.1% had heard of the concept of safe abortion. Of those, 57% have heard about safe abortion from mass media and 23.4% from their doctor. However, 253 of 502 participants (50.4%) of women were unaware of medication pills (mifepristone and misoprostol) safe methods in abortion. Additionally, 353 of 502 participants (70.3%) of the participants reported not being aware of the Lebanese abortion law and 26.3% knew that abortion is only legal if it endangers the life of the woman and 38.2% of the participants thought that contraceptive methods do not decrease abortion rates.

The mean knowledge score of participants was 9.76 ± 1.71, showing a moderate knowledge level (Table 2). Higher knowledge was significantly associated with living in urban areas, having a university degree or HE, medical education or working in medical field. All other sociodemographic factors were not significantly associated with knowledge (Supporting Information Table S1). When computing in multivariate logistic regression model, only working in the medical field remained independently associated with higher abortion knowledge scores (OR = 2.80, 95% CI: 1.04–7.56, *p* = 0.042), while all other factors previously associated were no longer statistical significant after adjustment (Table 3). Nearly half of participants considered Mifepristone and Misoprostol medication as safe abortion, which was significantly associated with the region, marital status, having children, occupation, major of study, socioeconomic status, and parents' educational level (Supporting Information Table S2). No sociodemographic factor was independently associated with good knowledge of the safety of medication abortion in the multivariate analysis, with the exception of paternal elementary education, which was shown to be associated with lower odds of correct knowledge compared with having an uneducated father (OR = 0.42, 95% CI: 0.19–0.92, *p* = 0.035) (Table 4).

**TABLE 2:** Knowledge of Lebanese women population on abortion. S.D. is the standard deviation.

Knowledge score	Mean ± S.D.
	9.76 ± 1.71

Knowledge assessment (of 502 participants)	Number (*n*)	Percentage (%)
Concept of abortion		
Familiar	475	94.6
Unfamiliar	27	5.4
Concept of safe abortion		
Familiar	317	63.1
Unfamiliar	185	36.9
Where respondents have heard about safe abortion (388)		
School	18	4.6
Parents	17	4.3
Mass media	221	57
Doctor or health care provider	91	23.4
Others	41	10.7
Perception of medication abortion (taking mifepristone and misoprostol)		
Unsafe abortion	253	50.4
Safe abortion	249	49.6
Perception of drinking toxic fluids to attempt abortion.		
Unsafe abortion	499	99.4
Safe abortion	3	0.6
Perception of surgical abortion		
Unsafe abortion	92	18.3
Safe abortion	410	81.7
Perception of inserting foreign objects into the uterus as a method of abortion		
Unsafe abortion	488	97.2
Safe abortion	14	2.8
Perception of applying pressure or trauma to the abdomen as a method of abortion		
Unsafe abortion	488	97.2
Safe abortion	14	2.8
Knowledge of the names of medication pills that cause abortion.		
No	359	71.5
Yes	143	28.5
Perception of the risk of abortion on a		
woman's reproductive health and her fertility	214	42.6
Not risky/not sure	288	57.4
Carries this risk		
Awareness of Lebanese abortion law		
Unaware	353	70.3
Aware	149	29.7
Perception of the legality of abortion		
Not legal, Legal in cases of rape or incest, Legal for any reason…	370	73.7
Legal if pregnancy endangers the life of the woman	132	26.3
Perception of the decision‐making that would permit legal safe abortion.		
Woman's decision/Attending doctor's approval/	241	48.0
From the approval of multiple doctors	261	52.0
Perception of the ability of contraceptive methods in decreasing abortion rates		
Doesn't affect abortion.	192	38.2
Decreases abortion rates	310	61.8
Knowledge of the risks of unsafe abortion: bleeding, death, infertility, and infections		
Unaware	9	1.8
Aware	493	98.2

**TABLE 3:** Multivariate logistic regression analysis for knowledge score.

Variable	AOR	95% CI	*p*‐value
Urban region	0.84	0.56–1.26	0.399
University education	0.86	0.49–1.50	0.589
Medical major	1.02	0.64–1.65	0.920
Working in medical field	2.80	1.04–7.56	**0.042**

*Note:* bold value indicate statically significant.

**TABLE 4:** Multivariate analysis of variables associated with good knowledge about safety of medication used in abortion.

Predictor	OR	95% CI	*p*‐value
Urban vs. rural	1.22	0.82–1.81	0.343
Having children vs. no children	1.09	0.48–2.46	0.853
Medical major vs. non‐medical	1.45	0.89–2.36	0.147
Employed vs. non‐employed	1.15	0.59–2.26	0.688
Student vs. non‐employed	1.06	0.64–1.77	0.812
Divorced vs. married	1.55	0.53–4.57	0.433
In relationship vs. married	1.52	0.54–4.25	0.422
Single, never in relationship vs. married	1.38	0.53–3.60	0.515
Single, no current relationship vs. married	0.84	0.32–2.22	0.737
Widowed vs. married	1.22	0.15–9.78	0.853
Middle SES vs. low SES	4.72	0.80–27.86	0.091
High SES vs. low SES	4.67	0.67–32.34	0.121
Mother elementary vs. uneducated	0.77	0.12–4.90	0.794
Mother middle school vs. uneducated	1.85	0.24–14.45	0.561
Mother high school vs. uneducated	0.77	0.12–5.12	0.791
Mother university vs. uneducated	0.38	0.06–2.39	0.304
Father elementary vs. uneducated	0.42	0.19–0.92	**0.035**
Father middle school vs. uneducated	0.56	0.25–1.27	0.163
Father high school vs. uneducated	0.61	0.28–1.33	0.212
Father university vs. uneducated	0.98	0.43–2.24	0.961

*Note:* bold value indicate statically significant.

Regarding the participants' attitudes toward abortion, it was found that 310 of 502 participants (61.8%) thought that abortion should not be socially acceptable and only 35.7% thought that abortion should be generally available. Of the participants, 55.2% reported knowing and trusting a healthcare worker who provides safe access to abortion. Interestingly, half of the participants would consider doing an abortion but depending on the situation (Table 5). Positive abortion social acceptability among participants was significantly associated with marital status, having children, place of residence, educational level, occupational status, and their parents' educational level (Supporting Information Table S3). In the multivariate logistic regression, both being married and residence in Beirut was independently associated with lower odds of supporting abortion social acceptability (OR = 0.48, 95% CI: 0.24–0.97, *p*= 0.041) and (OR = 0.52, 95% CI: 0.31–0.85, *p*= 0.009), respectively. In contrast, higher odds of supporting abortion social acceptability were independently associated with having an educated mother (OR = 3.52, 95% CI: 1.98–6.25, *p*< 0.001) (Table 6).

**TABLE 5:** Attitudes of participants regarding abortion.

Attitude assessment (of 502 participants)	Number (*n*)	Percentage (%)
Abortion should be more socially acceptable.		
Yes	192	38.2
No	310	61.8
Abortion should be.		
Not available	90	17.93
Difficult to obtain	233	46.41
Generally available	179	35.66
Know and trust a healthcare worker that provides access to safe abortion.		
Yes	275	55.2
No	227	44.8
Encourage a woman with an unwanted pregnancy to do an abortion.		
No	206	41.0
Yes/Leave it up to her	296	59
Undergoing abortion is		
Women's decision	55	10.9
Partner's decision	9	1.8
Both decision	438	87.3
Know someone who's had an induced abortion.		
No	247	49.2
A family member	113	22.5
Friend	58	11.6
Others	84	16.7
Consider undergoing an induced abortion in case of an unwanted pregnancy.		
Yes	67	13.3
No	185	36.9
Depends on the situation	250	49.8

**TABLE 6:** Multivariate analysis for positive attitude toward abortion social acceptability.

Variable	OR	95% CI	*p*‐value
Married	0.48	0.24–0.97	**0.041**
Having children	1.11	0.54–2.26	0.775
Beirut residency	0.51	0.31–0.85	**0.009**
University education	1.53	0.82–2.86	0.186
Student	0.91	0.51–1.60	0.732
Employed	0.65	0.32–1.31	0.228
Father university educated	1.59	1.00–2.53	0.051
Mother university educated	3.52	1.98–6.25	**< 0.001**

*Note:* bold values are statically significant.

Regarding the practice of abortion, 14.7% of the participants reported experiencing a spontaneous abortion and 14.3% of them experienced an unwanted pregnancy. A key finding is 45 of 502 participants (9.0%) performed an induced abortion. Among the 45 participants who reported having undergone induced abortion, 40 of the 45 participants (88.9%) reported using medically safe methods, including surgical abortion (66.7%) or medication abortion (22.2%). This finding is notable given Lebanon's restrictive legal framework and suggests a discrepancy between legal restrictions and actual access to abortion services. Regarding the decision for abortion, 46.7% of them did the procedure following their doctor's advice and 40% of them made the decision completely on their own (Table 7). When computed in multivariate logistic regression model, having children (OR = 3.21, 95% CI: 1.02–10.08, *p* = 0.046), use of implants/IUD (OR = 2.94, 95% CI: 1.29–6.71, *p* = 0.010), and condom use (OR = 2.66, 95% CI: 1.29–6.71, *p* = 0.029) are independently associated with higher odds of reporting induced abortion practice after adjustment (Table 8).

**TABLE 7:** Reported practice of abortion among participants.

Practice assessment (of 502 participants)	Number (*n*)	Percentage (%)
Number of pregnancies		
0	262	52.1
1	42	8.3
2	60	12.0
3	53	10.6
4	51	10.2
5	18	3.6
> 5	16	3.2
Visiting a gynecologist		
Once every 6 months	65	12.9
Every year	95	18.9
Every 2 years	44	8.8
Rarely ever	298	59.4
Experienced an unwanted pregnancy		
Yes	72	14.3
No	430	85.7
Experienced a spontaneous abortion.		
Yes	74	14.7
No	428	85.3
Number of spontaneous abortions experienced (167)		
1	101	60.4
2	49	29.34
3	14	8.38
4	1	0.59
5	2	1.19
Contraceptive method(s) of choice (468)		
Medication pills	26	5.56
Implants and IUD	41	8.76
Condoms	27	5.77
Others	50	10.68
	324	69.23
Done an induced abortion.		
Yes	45	9.0
No	457	91.0
Experience of any problem after undergoing abortion (45)		
Yes	7	15.6
No	38	84.4
Influence of the decision to terminate the pregnancy (45)		
You took this decision completely on your own.	18	40.0
Doctor's advice	21	46.7
Your partner	6	13.3
Abortion method practiced (45)		
Medication abortion	10	22.2
Surgical abortion	30	66.7
Drinking toxic fluids	1	2.2
Other	4	8.9
Reason(s) for terminating pregnancy (86)		
Financial reasons	12	26.67
Reached desired number of children.	8	17.78
Outside the preferred age to be pregnant.	3	6.67
Social reasons (unmarried, divorced, to continue her studies)	9	20
High‐risk pregnancy (dangerous for the woman)	22	48.89
Karyotype shows that the fetus is deformed or carries a genetic abnormality. Or in case the fetus has a low chance of survival.	13	28.89
Rape	2	4.44
Unwanted pregnancy	17	37.78

**TABLE 8:** Multivariate logistic regression analysis for induced abortion practice.

Predictor	OR	95% CI	*p*‐value
Having children	3.21	1.02–10.08	**0.046**
Student vs. employed	1.50	0.43–5.22	0.524
Non‐employed vs. employed	1.31	0.33–5.27	0.699
University education vs. below university	0.65	0.27–1.57	0.343
South vs. North Lebanon	0.62	0.14–2.69	0.520
Beirut vs. North Lebanon	2.01	0.55–7.34	0.290
Beqaa vs. North Lebanon	2.05	0.62–6.84	0.242
Mount Lebanon vs. North Lebanon	1.90	0.68–5.32	0.222
Medication pills contraceptive use	1.27	0.51–3.18	0.612
Implants/IUD use	2.94	1.29–6.71	**0.01**
Condom use	2.66	1.11–6.40	**0.029**
Other contraceptive use	2.02	0.89–4.61	0.094

*Note:* bold values are statically significant.

Induced abortion practice was associated with place of residence, marital status, having children, occupation, educational level, socioeconomic status, and contraceptive use (*p* < 0.05). However, working in the medical field or major of study was not associated with induced abortion practice (Supporting Information Table S4).

## 4 Discussion

This study showed that although a moderate knowledge (mean score of 69.7%) regarding abortion existed among the Lebanese women, a gap remained regarding the medication abortion and its legal aspect. Participants generally were able to recognize unsafe abortion practices, yet only half were able to correctly identify mifepristone and misoprostol as safe abortion practices. Thus, these findings may suggest a greater understanding for the unsafe abortion risks than for the care options of evidence‐based abortion. The moderate overall knowledge observed can be partially explained by the emergence of the health‐related information distrusted through mass media which was reported as the major source of information regarding abortion. Similar findings were previously reported in Nepal and in Ethiopia [14, 19]. Yet, mass media has a potential drawback as information can be scattered, fragmented and subjective regarding the legal aspect of abortion and medical abortion methods which may suggest the presence of moderate knowledge while substantial level of misconception existed regarding the medication and the Lebanese abortion law. Moreover, medical education may potentially contribute to the knowledge as participants with medical education background showed higher knowledge scores in bivariate analyses. Greater exposure to reproductive and maternal health and evidence‐based clinical practice regarding abortion are part of the medical curricula enhancing participants' understanding of abortion safety and risks. In the multivariate model, working in the medical field remained significant predictor of higher knowledge suggesting a role of health‐care related professional exposure in increasing the knowledge most likely by encountering health information, patient counseling and clinical guidelines frequently. Thus, facilitating access to authentic reproductive health information among general information is crucial to improve their knowledge and safe practice.

Almost three‐quarters of participants were unaware of Lebanon's abortion law, considering it a criminal act unless to save the mother's life. This might be due to the absence of reproductive health education and ironic fact that safe abortion is widely available despite legislative restrictions may be the cause of this disparity.

Despite participants showed moderate knowledge, their awareness was considered low. In fact, two‐thirds of participants were unaware of the legal aspect governing abortion which may suggest a discrepancy between legal literacy and reproductive health information.

As for the factors influencing the attitude toward abortion, an association was found between marital status and the perception of abortion's social acceptability. The conservative attitude held by married women might be due to marriage security providing protection against abortion. Similarly, women who have no sexual experience might be against abortion due to the lack of personal encounters with the complexities of unintended pregnancy as already observed in another study in Lao PDR [20]. Interestingly, women's opinions toward abortion were significantly impacted by parental education. Abortion acceptance was positively correlated with both mother and father education levels demonstrating that in conservative communities, intergenerational schooling affects reproductive autonomy. This association can be partly explained by theories of social transmission and intergenerational socialization. In Lebanon, parents have a central role in a child's beliefs and personality, value, and perspectives. Critical thinking and engagement with diverse perspectives and evidence‐based information might be encouraged more from parents with higher educational background. Thus, their children may have more developed perspectives regarding complex social stigma, including abortion. Maternal education remained independently associated with abortion attitudes supporting the fact that family social level contributes to the child's views regarding reproductive acceptability and rights.

Although all unsafe abortions were attributed to developing countries [1], 9% of Lebanese women reported having safe induced abortion despite its legal framework [17], which explains the nonconformity with the general fact set by WHO [1]. Similar findings were observed in southwest Ethiopia [21].

The observed limited awareness of abortion law among participants may be partly reflected by limited and insufficient sexual and reproductive health education within schools and universities. Many programs focus on the biological aspects of reproductive health with limited information regarding the reproductive rights and legal framework. Women might not develop an adequate awareness regarding the legal framework, although they acquired enough general information about abortion. Moreover, abortion is socially sensitive in Lebanon and still considered as a stigmatized topic avoided in familial or educational discussions, thus, discouraging women from seeking information and resulting in misinformation and uncertainty.

Findings need to be interpreted carefully due to sampling technique. Participants were recruited conveniently through online recruitment, which may include sampling bias toward reaching more women with internet access and higher educational background. Those participants may have greater access to health information than the general population.

These findings suggest that legal restriction is not the only challenge related to abortion in Lebanon. Persistent social stigmatization, low awareness in legal framework and unequal access to health information, more specifically, reproductive and sexual information may be considered as predictors for abortion misconceptions and barriers among Lebanese women. Therefore, stakeholders should focus more on public health initiatives to improve legal literacy not only increasing health education and to reduce burden limiting equal access to evidence‐based information regarding abortion.

## 5 Limitations

Several limitations might affect the generalization of these findings. This is a cross‐sectional study with no cause‐effect relation between sociodemographic characteristics and abortion‐related knowledge, attitudes, and practices. Participants were recruited through an online approach which may introduce selection bias resulting in overrepresentation of women with greater exposure to health information. A possible social bias and underreporting of abortion practices might exist due to the stigma nature of abortion in Lebanon. Finally, participants self‐reported their information which might introduce recall bias.

## Author Contributions

**Youssef Abou Dib:** conceptualization, investigation, methodology, validation, software, project administration, writing – original draft, writing – review and editing. **Rayyan Hammoud:** conceptualization, writing – original draft, writing – review and editing, methodology. **Sara Al Smaily:** conceptualization, writing – original draft, methodology, writing – review and editing. **Tarek Abdul Samad:** methodology, writing – review and editing, writing – original draft, conceptualization. **Nour Mina:** conceptualization, writing – original draft, methodology, writing – review and editing. **Aline Bou Dargham:** conceptualization, writing – original draft, writing – review and editing, methodology. **Alaa Abou Saleh:** conceptualization, methodology, writing – review and editing, writing – original draft. **Bilal Azakir:** conceptualization, investigation, writing – original draft, methodology, validation, visualization, writing – review and editing, formal analysis, project administration, supervision, data curation, resources, software, funding acquisition.

## Funding

The authors have nothing to report.

## Conflicts of Interest

The authors declare no conflicts of interest.

## Policy on Using ChatGPT and Similar AI Tools

The authors used ChatGPT (OpenAI, San Francisco, CA, USA) to assist with language editing, grammar refinement, and improvement of manuscript readability during manuscript preparation and revision.

## Transparency Statement

Bilal Azakir affirms that this manuscript is an honest, accurate, and transparent account of the study being reported; that no important aspects of the study have been omitted; and that any discrepancies from the study as planned have been explained.

## Supporting information


**Table S1:** Association between sociodemographic information and knowledge score.
**Table S2:** Association between sociodemographic information and taking mifepristone and misoprostol medication.
**Table S3:** Associations between sociodemographic and attitudes of participants regarding abortion.
**Table S4:** Associations between sociodemographic information and the practice of induced abortion.

## Data Availability

The data that support the findings of this study are available from the corresponding author upon reasonable request.
