# Supplementary material for: Knowledge and Attitude of Lebanese Women Regarding Induced Abortion: A Cross‐Sectional Study
Source: Health Sci Rep. 2026 Aug 11;9(8):e73027. doi: 10.1002/hsr2.73027 (PMC13462664; doi:10.1002/hsr2.73027)
Supplement: Supplementary file 1 [file HSR2-9-e73027-s001.docx]

**Supplementary table 1**: Association between sociodemographic information and knowledge score.

|  | Mean ± SD | *p* value |
| --- | --- | --- |
| KNOWLEDGE SCORE  (14 questions) | **9.76 ± 1.71** |  |
| Marital status  Single, never been in a relationship.  Single, no current relationship  In a relationship  Married  Divorced  Widowed | 9.87±1.83  9.58±1.69  10.21±1.92  9.68±1.57  9.70±1.72  8.75±1.70 | .206 |
| Children  Yes  No | 9.73±1.57  9.78±1.80 | .744 |
| Residency  North Lebanon  Mount Lebanon  Beqaa  South Lebanon  Beirut | 9.56±1.61  9.86±1.48  9.68±1.77  9.64±2.03  10.21±1.79 | .204 |
| Region  Urban  Rural | 10.08±1.65  9.53±1.70 | .000* |
| Level of education  Uneducated  High school  University and HE | 9.81±1.45  9.84±2.29  10.33±1.77 | .001* |
| Field of education  Medical  Non-medical | 10.52±1.63  9.43±1.57 | .000* |
| Occupation  Employed  Student  Non-employed | 9.84±1.73  9.67±1.75  9.74±1.60 | .632 |
| Work field  Medical  Non-medical | 10.82±1.37  9.35±1.71 | .000* |
| Religious Status:  Yes  No  To a certain extent | 9.73±1.58  9.80±1.72  9.75±1.66 | .927 |

**Supplementary table 2:** Association between sociodemographic information and taking mifepristone and misoprostol medication.

| Taking mifepristone and misoprostol (of 502 participants) | Unsafe abortion | Safe abortion | P Value |
| --- | --- | --- | --- |
| Characteristics: | Number (percentage) | Number (percentage) |  |
| Total participants | 249 (49.6) | 253 (50.4%) |  |
| Place of residency  South  Beirut  Beqaa  Mount Lebanon  North Lebanon | 46 (46.9%)  17 (36.2%)  40 (54.8%)  95 (54.9%)  55 (49.5%) | 52 (53.1%)  30 (63.8%)  33 (45.2%)  78 (45.1%)  56 (50.5%) | .179 |
| Region  Urban  Rural | 90 (43.5%)  163 (55.2%) | 117 (56.5%)  132 (44.8%) | .009* |
| Marital status:  Single, never been in a relationship.  Single, no current relationship  In a relationship  Married  Divorced  Widowed | 42 (38.2%)  41 (48.8%)  20 (36.4%)  141 (60.8%)  7 (41.2%)  2(50%) | 68 (61.8%)  43 (51.2%)  35 (63.6%)  91 (39.2%)  10 (58.8%)  2 (50%) | $.001$* |
| Have children.  Yes  No | 133 (59.1%)  120 (43.2%) | 92 (40.9%)  157 (56.8%) | .000* |
| Occupation  Employed  Student  Unemployed | 77 (40.3%)  96 (53.0%)  80 (61.5%) | 114 (59.7%)  85 (47.0%)  50 (38.5%) | .001* |
| Work in the medical field: (181) **  Yes  No | 16 (40.0%)  80 (56.7%) | 24 (60.0%)  61 (43.3%) | .061 |
| Educational Level  Elementary school  High School  University | 14 (53.8%)  35 (61.4%)  204 (48.7%) | 12 (46.2%)  22 (38.6%)  215 (51.3%) | .333 |
| Major of study: (419) **  Medical  Non-medical | 53 (34.6%)  151 (56.8%) | 100 (65.4%)  115 (43.2%) | .000* |
| Perceived socioeconomic status.  Low class  Middle class  High class | 27 (73.0%)  221 (49.5%)  5 (26.3%) | 10 (27.0%)  225 (50.5%)  14 (73.7%) | .002* |
| Mother’s Educational Status  Uneducated  Elementary School  Middle school  High School  University and HE | 24 (68.6%)  185 (58.2%)  5 (20.0%)  15 (34.1%)  24 (30.0%) | 11 (31.4%)  133 (41.8%)  20 (80.0%)  29 (65.9%)  56 (70.0%) | .000* |
| Father’s Educational Status  Uneducated  Elementary School  Middle school  High school  University and HE | 19 (50.0%)  79 (65.3%)  62 (53.4%)  51 (52.0%)  42 (32.5%) | 19 (50.0%)  42 (34.7%)  54 (46.6%)  47 (48.0%)  87 (67.5%) | .000* |

**Supplementary table 3:** Associations between sociodemographic and attitudes of participants regarding abortion.

| Do you think abortion should be more socially acceptable | Yes | No | *p* Value |
| --- | --- | --- | --- |
| Characteristics: | Number (percentage) | Number (percentage) |  |
| Marital status  Single, never been in a relation.  Single, no current relation.  In a relation  Married  Divorced  Widowed | 44 (40)  44 (52.3)  27 (49.1)  66 (28.5)  11(64.7)  0 (0) | 66 (60)  40 (47.6)  28 (50.9)  166 (71.5)  6 (35.3)  4 (100) | .000* |
| Do you have any children?  Yes  No | 68 (30.2)  124 (52.3) | 157 (69.8)  153 (47.6) | .001* |
| Residency  North Lebanon  Mount Lebanon  Beqaa  South Lebanon  Beirut | 33 (29.7)  82 (47.4))  26 (35.6)  32 (32.6)  19 (40.4) | 78 (70.3)  91 (52.6)  47 (64.4)  66 (67.2)  28 (59.6) | .025* |
| Region  Urban  Rural | 79 (38.2)  113 (38.3) | 128 (61.8)  182 (61.7) | .975 |
| Level of education  Uneducated  High school  University and HE | 4 (15.4)  17 (29.8)  171 (40.8) | 22 (84.6)  40 (70.2)  248 (59.2) | .028* |
| Field of education (419) **  Medical  Non-medical | 71 (46.4)  100 (37.6) | 82 (53.6)  166 (62.4) | .077 |
| Occupation  Employed  Student  Non-employed | 83 (43.4)  72 (39.8)  37 (28.5) | 108 (56.6)  109 (60.2)  93 (71.5) | .022* |
| Work field (181) **  Medical  Non-medical | 16 (40)  56 (39.7) | 24 (60)  85 (60.3) | .974 |
| Father’s educational status  Uneducated  Elementary school  Middle school  High school  University and higher education | 9 (23.7%)  31 (25.6%)  43 (37.1%)  38 (38.8%)  71 (55%) | 29 (76.3%)  90 (74.4%)  73 (62.9%)  60 (61.2%)  58 (45%) | .000* |
| Mother’s educational status  Uneducated  Elementary school  Middle school  High school  University and higher education | 11 (31.4%)  87 (27.3%)  12 (48%)  27 (61.4%)  55 (68.7%) | 24 (68.6%)  231 (72.2%)  13 (52%)  17 (38.6%)  25 (31.3%) | .000* |

**Supplementary table 4:** Associations between sociodemographic information and the practice of induced abortion.

| Done an induced abortion (of 502 participants) | Yes | No | p Value |
| --- | --- | --- | --- |
| Characteristics: | Number (percentage) | Number (percentage) |  |
| Place of residency  South  Beirut  Beqaa  Mount Lebanon  North Lebanon | 3 (3.06%)  6 (12.77%)  7 (9.59%)  23 (13.29%)  6 (5.41%) | 95 (96.94%)  41 (87.23%)  66 (90%)  150 (86.71%)  105 (94.59%) | .03* |
| Marital status:  Single, never been in a relationship.  Single, no current relationship  In a relationship  Married  Divorced  Widowed | 1(0.91%)  2 (0.88%)  3 (2.13%)  34 (7.01%)  4 (14.81%)  1 (8.33%) | 109 (99.09%)  224 (99.12%)  138 (97.87%)  451 (92.99%)  23 (91.9%)  11 (91.67%) | $.000$* |
| Have children  Yes  No | 38 (16.89%)  7 (2.53%) | 187 (83.11%)  270 (97.47%) | .000* |
| Occupation  Employed  Student  Unemployed | 5 (2.62%)  22 (12.15%)  18 (14.18%) | 186 (97.38%)  159 (87.85%)  112 (86.15%) | .000* |
| Work in the medical field: (181) **  Yes  No | 2 (5%)  20 (90.9%) | 38 (95%)  121 (85.82%) | .117 |
| Educational Level  Elementary school  High School  University | 1 (2.22%)  13 (28.9%)  31 (20.0%) | 4 (97.78%)  66 (71.1%)  388(68.89%) | .04* |
| Major of study: (419) **  Medical  Non-medical | 7 (22.6%)  24 (%) | 146 (93%)  242 (22.6%) | .094 |
| Perceived socioeconomic status.  Low class  Middle class  High class | 10 (22.2%)  35 (77.8%)  0 (0.0%) | 27 (77.8%)  411 (22.2%)  19 (100%) | .000* |
| Use of Medication contraceptives ***  Yes  No | 8 (17.78%)  37 (82.22%) | 44 (82.22%)  413 (17.78%) | .000* |
| Use of implants and IUDs  Yes  No | 10 (22.22%)  30 (66.67%) | 54 (77.78%)  403 (33.33%) | .000* |
| Use of Condoms  Yes  No | 7 (15.56%)  38 (84.44%) | 24 (84.44%)  433 (15.56%) | $.000$* |
| Use of other contraceptives  Yes  No | 20 (44.44%)  25 (55.55%) | 355 (55.56%)  102 (44.45%) | $.000$* |

| Perceived socioeconomic status.  Low class  Middle class  High class | 10 (27)  172 (38.6)  10 (52.6) | 27 (73)  274 (61.4)  9 (47.4) | .161 |
| --- | --- | --- | --- |
